# Irritable Bowel Syndrome: Clinical Manifestations, Dietary Influences, and Management

**DOI:** 10.3390/healthcare5020021

**Published:** 2017-04-26

**Authors:** Ronald Ikechi, Bradford D. Fischer, Joshua DeSipio, Sangita Phadtare

**Affiliations:** 1 Department of Biomedical Sciences, Cooper Medical School of Rowan University, Camden, NJ 08103, USA; ikechir0@rowan.edu (R.I.); fischerb@rowan.edu (B.D.F.); 2 Department of Medicine, Gastroenterology/Liver Diseases Division, Cooper Medical School of Rowan University, Camden, NJ 08103, USA; DeSipio-Joshua@CooperHealth.edu

**Keywords:** irritable bowel syndrome (IBS), fructose, high-fructose corn syrup (HFCS), functional gastrointestinal disorder

## Abstract

Irritable bowel syndrome (IBS) is a functional gastrointestinal disorder that is characterized by symptoms of chronic abdominal pain and altered bowel habits in the absence of an overtly identifiable cause. It is the most commonly diagnosed functional gastrointestinal disorder, accounting for about one third of gastroenterology visits. It generally presents as a complex of symptoms, including psychological dysfunction. Hypersensitivity to certain foods, especially foods that contain high amounts of fructose, plays a role in the pathophysiology of IBS. Elevated consumption of high-fructose corn syrup (HFCS) has been discussed in this aspect. The treatment options for IBS are challenging and varied. In addition to dietary restrictions for HFCS-induced IBS, such as low-FODMAP (Fermentable Oligosaccharides, Disaccharide, Monosaccharides, and Polyols) diets, existing drug therapies are administered based on the predominant symptoms and IBS**-**subtype. Patients with IBS are likely to suffer from issues, such as anxiety, depression, and post-traumatic-stress disorder. Biopsychosocial factors particularly socioeconomic status, sex, and race should, thus, be considered for diagnostic evaluation of patients with IBS.

## 1. Introduction

Irritable bowel syndrome (IBS) is the most commonly diagnosed functional gastrointestinal disorder characterized by chronic abdominal pain and changed bowel habits in the absence of an overtly identifiable cause. Although regional variation exists, the prevalence of IBS ranges from 10–15% in population-based studies in North America and Europe [1,2,3,4,5]. Annually, in the United States, there are about 3.1 million ambulatory office visits and total expenditures exceeding $20 billion [6,7]. Geographic variations range from 7% in South Asia to 21% in South America. The prevalence of IBS is most common between 20 and 40 years of age with a significant female predominance [8].

IBS has gained significant interest in healthcare due to its high occurrence, complex pathophysiology, difficult diagnosis due to a wide range of non-specific symptoms, and varied and challenging treatment options. In addition to these aspects, this review also discusses the dietary influences on the development of IBS, for example, consumption of high amounts of fructose especially while using refined products such as high-fructose corn syrup (HFCS), along with the biopsychosocial aspects of IBS. The recent reports that link IBS to psychiatric co-morbidities, including post-traumatic stress disorder, anxiety, and depression, especially in low-income areas with underserved populations, are of particular concern. This article also touches upon observations made for the patients with IBS in our local community.

## 2. Presentations of Symptoms in IBS

IBS can present with a wide range of both gastrointestinal and extraintestinal symptoms. These include (i) chronic abdominal pain with variable intensity and periodic exacerbations; (ii) altered bowel habits ranging from diarrhea, constipation, alternating diarrhea and constipation, or normal bowel habits alternating with either diarrhea and/or constipation; (iii) diarrhea characterized by frequent loose stools of small to moderate volume; (iv) prolonged constipation with interludes of diarrhea or normal bowel function with often hard, pellet-shaped stools and a sense of incomplete evacuation even when the rectum is empty; and (v) extraintestinal symptoms, such as impaired sexual function, dysmenorrhea, dyspareunia, increased urinary frequency and urgency, and fibromyalgia symptoms [9].

## 3. Defining IBS Using a Diagnostic Approach

The definition of IBS has been evolving since the first Rome I guidelines for IBS was released in 1989. The most recent Rome IV criteria released in May of 2016 shifts our understanding from the “absence of structural disease” to a “disorder of gastrointestinal functioning”. Rome IV expanded upon this concept of disorders of gut-brain interaction as it is related to motility disturbance, visceral hypersensitivity, altered mucosal and immune function, altered gut microbiota, and altered central nervous system (CNS) processing.

Broadly speaking, based on the Rome criteria, IBS is defined as recurrent abdominal pain associated with altered defecation. Four subtypes of IBS are recognized:

(1) Constipation predominant IBS (hard or lumpy stools ≥25%/loose or watery stools <25% of bowel movements);
(2) Diarrhea predominant IBS (loose or water stools ≥25%/hard or lumpy stools <5% of bowel movements);
(3) Mixed IBS (hard or lumpy stools ≥25%/loose or watery stools ≥25% of bowel movements); and
(4) Unsubtyped IBS (insufficient abnormality of stool consistency to meet the above subtypes).

The symptoms of IBS are not distinguishable from those of several other gastrointestinal disorders. This makes its diagnosis difficult. A variety of methods [10,11,12], ranging from manometry, colonoscopy, and enteroclysis, along with stool cultures and blood tests, may prove to be useful for the exclusion of organic disease. Routine laboratory studies, such as complete blood count and blood enzyme panels, are normal in IBS. The diagnostic evaluation depends upon whether the predominant symptom is diarrhea or constipation. The patient’s clinical history including stool cultures, screening of celiac disease, and colonoscopy are important in IBS characterized predominantly by diarrhea, while clinical history including radiography, flexible sigmoidoscopy, and colonoscopy are important in IBS with predominant constipation. Patients with constipation-predominant or diarrhea-predominant IBS tend to have decreased or increased colonic contractions and transit rates, respectively. Psychological factors and dietary habits are also used as screening tools for IBS.

## 4. Pathophysiology of IBS

Pathophysiology of IBS is complex and is considered to be due to combination of several factors, such as (i) motor abnormalities, such as increased frequency and irregularity of luminal contractions, prolonged transit time with constipation predominance, and an exaggerated motor response to cholecystokinin and meal ingestion with prominent diarrhea symptoms; (ii) increased sensation in various receptors in the gut wall in response to stimuli leading to distention and bloating; (iii) activation of the mucosal immune system characterized by alterations in particular immune cells and markers leading to increased release of nitric oxide, histamine, and proteases. These, in turn, stimulate the enteric nervous system and lead to abnormal motor and visceral responses within the intestine, (iv) increased risk associated with viral, bacterial, protozoan, and helminth infections; (v) malabsorption, for example, due to bile acid malabsorption, as a result of enteric infections; (vi) increase in serotonin-containing enteroendocrine cells and T lymphocytes following acute *Campylobacter enteritis*. This leads to increased gastrointestinal motility and visceral hypersensitivity; (vii) antibiotic use; (viii) changes in gut microflora; (ix) small intestinal bacterial overgrowth (SIBO); (x) sensitivity to certain foods such as fructose intolerance and gluten sensitivity; (xi) polymorphisms in the serotonin transporter gene that may result in changed serotonin reuptake efficacy which, in turn, influences intestinal peristalsis; and (xii) stress, increased anxiety, depression, and phobias [9].

## 5. Dietary Influences on Symptoms Seen in Patients with IBS

### 5.1. Fructose

As mentioned above, the pathophysiology of IBS is not fully understood. One of the contributing factors is hypersensitivity to certain foods. Although well-defined food allergies are not common in patients with IBS, exacerbation of IBS symptoms has been observed due to fructose malabsorption [13]. Fructose is a six-carbon monosaccharide. It is naturally present in a variety of foods, for example, fruits, vegetables, and honey. It is also enzymatically produced from corn as HFCS, which is commonly found in many food sweeteners and soft drinks. Fructose is found in three main forms in the diet: as free fructose (present in fruits and honey); as a constituent of the disaccharide sucrose; or as fructans, a polymer of fructose, usually in oligosaccharide form (present in some vegetables and wheat) [14]. Examples of some of the foods that contain high amounts of fructose are shown in Table 1.

Approximately, one third of patients with suspected IBS have fructose malabsorption and dietary fructose intolerance. Fructose absorption is not highly efficient as it is energy independent. While glucose is completely absorbed through an active transport mechanism facilitated by GLUT-2 and GLUT-5 transporters, fructose is mainly absorbed through carrier-mediated facilitative diffusion and GLUT-5. Even small magnitude increases in the dietary fructose can overwhelm the transporter capacity. Malabsorption of fructose leads to water influx into the lumen due to osmotic pressure. This, in turn, results in rapid propulsion of bowel contents into the colon. Unabsorbed fructose is fermented by colonic bacteria, which results in the production of short-chain fatty acids, hydrogen, carbon dioxide, and trace gases. This can result in symptoms including abdominal pain, excessive gas, and bloating. Hydrogen must be excreted in breath and flatus as it cannot be metabolized by humans. A rise in breath hydrogen (and/or methane) following substrate ingestion is the basis for detecting incomplete fructose absorption and estimating fructose absorptive capacity. Fructose malabsorption can, thus, be identified by a positive breath test, which is most commonly defined as a rise in hydrogen and/or methane (methane is less frequently measured) of at least 20 ppm (less often 10 ppm for methane) peaking from 1.5 to 3 h after ingestion of the test carbohydrate. Studies have shown that, in some individuals, consumption of as little as 5 g fructose may lead to malabsorption issues. In a randomized, double-blind, dose-response study, healthy individuals were able to tolerate 25 g of fructose, but when the amounts of fructose were doubled, 80% of patients exhibited malabsorption as measured by the breath analysis. Fifty percent of these patients suffered from mild to moderate belching, bloating, or diarrhea [15]. Several mechanisms have been proposed to explain how unabsorbed fructose may lead to symptoms seen in IBS, such as (i) osmotic effect, leading to an increase in the gastrointestinal motility; (ii) bacterial fermentation of unabsorbed fructose leading to the production of short chain fatty acids, hydrogen, carbon dioxide, and trace gases; and (iii) local irritation caused due to physical contact of fructose with the intestinal tract [13].

Choi et al. specifically assessed fructose intolerance in patients suffering from IBS and long-term outcome of fructose-restricted diet in these patients. Two hundred and nine patients with IBS were retrospectively evaluated for organic illnesses. About one-third of patients with IBS had fructose intolerance. Patients with a positive fructose breath test received instructions regarding a fructose-restricted diet. One year later, their symptoms, compliance with, and effects of dietary modification on lifestyle were assessed using a structured interview. Symptoms improved on fructose-restricted diet in compliant patients, while noncompliance was associated with persistent symptoms [16]. In another double-blinded, randomized, quadruple arm, placebo-controlled re-challenge trial, 25 participating patients were provided foods that were low in free fructose and fructans. These patients had previously shown improvement in their IBS symptoms in response to dietary change. Patients were randomly challenged by graded dose introduction of fructose and fructans, alone or in combination, or glucose. Symptoms were monitored daily. It was observed that symptoms were induced in a dose-dependent manner in response to the inclusion of fructose and fructans and mimicked previous IBS symptoms [17]. As mentioned above, fructose absorption is coupled with that of glucose. In most of the fruits and vegetables, the fructose to glucose ratio is low and the problems associated with fructose malabsorption are not significant. Glucose increases fructose absorption in a dose-dependent manner. The efficacy of this increase depends on the proportion of glucose relative to fructose. A study using healthy subjects showed that an equimolar dose of glucose normalizes fructose absorption [18]. Glucose increases fructose absorption most probably by passive diffusion, but it is also possible that this facilitation of fructose absorption is due to the glucose-mediated delayed gastric emptying. Consistent with this, it is observed that fruits with high fructose to glucose concentrations, such as blueberries, pears, mangoes, papaya, apples, and watermelon, if consumed in high amount and in isolation, may lead to malabsorption problems, which are exacerbated in patients with IBS. Refined products that are particularly high in fructose relative to glucose (such as HFCS-90 or agave,) may particularly pose problems for patients with IBS [13].

Clinicians, while working with patients that have a diagnosis of IBS, spend a large portion of office visits in reviewing the dietary journals of their patients. Discussions include specific amounts of foods that are consumed and their relation to the patient’s overall symptoms. The FODMAP (Fermentable Oligosaccharides, Disaccharide, Monosaccharides, and Polyols) diet, which includes fructose, is usually part of these discussions due to the existing supportive evidence that it significantly improves IBS symptoms. Breath testing after ingestion of fructose has also been adopted as a standard method of identifying fructose malabsorption and intolerance. There are limitations to these tests; for example, a breath test may not be optimal in patients suffering from IBS that may also have small intestinal bacterial overgrowth. Optimal hydrogen breath test parameters are still unclear and reproducibility of this test is limited. Most studies do not screen for, or measure, methane production, potentially resulting in under-diagnosis. However, breath tests are the best available tools for diagnosis of fructose intolerance at present. Presence of malabsorption and reproduction of symptoms during a breath test provides the best objective evidence and symptom correlation for fructose intolerance that can then lead to a firm diagnosis, and this helps avoid the use of empirical or unnecessarily restrictive diets [19].

The Academy for Nutrition and Dietetics Guidelines for fructose intolerance include foods with less than 3 g of fructose per serving, less than 0.5 g of free fructose (defined as fructose in excess of glucose) per 100 g of food and less than 0.5 g of fructan per serving but these guidelines are only arbitrary cut-off values [20]. Fructose malabsorption is most strongly influenced by the free fructose content of the food, however, consumption of high amounts of total fructose can also result in the symptoms seen in the patients with IBS. Studies have shown that the breath hydrogen levels were four times higher when 50 g of free fructose was consumed as compared to when 50 g of fructose was consumed in the form of sucrose [21].

There are no established protocols or guidelines in the dietary management of fructose malabsorption or intolerance and, therefore, management depends on the providers’ experience. As mentioned above, dietary limitation is the most commonly adapted approach. This may also include an elimination phase, where patients are encouraged to follow a diet with approximately 5 g of fructose per day for about two weeks. A completely fructose-free diet is not practical to achieve and is not necessary. The elimination phase is followed by a re-introduction phase, where small amounts of slightly higher fructose-containing foods, one at a time, are allowed. This helps to determine exactly how much fructose is tolerated. Based on these approaches diets are designed that are minimally restrictive and allow for the management of symptoms [19]. Alternative approaches include the use of xylose isomerase, which converts fructose to glucose [22].

### 5.2. High Fructose Corn Syrup (HFCS)

High-fructose corn syrup (HFCS) is a disaccharide consisting of one molecule each of fructose and glucose. However, unlike sucrose, which is comprised of equal parts fructose and sucrose, HFCS has two main derivations, HFCS-55 and HFCS-42. High Fructose Corn Syrup-55 is made up of 55% fructose and 42% glucose, whereas HFCS-42 is made up of 55% glucose and 42% fructose. In HFCS-90 or agave syrup, fructose accounts for 84.29% of the carbohydrate content. HFCS can be found in its natural form in select fruits and vegetables and also in concentrated artificial forms, such as sweeteners. The introduction of high fructose corn syrups (HFCS) as alternative sweeteners to sucrose in the 1960s resulted in a dramatic increase in the monosaccharide form of fructose in the US food supply. HFCS remains the most widely used sweetener in beverages, dairy products, canned, baked, and processed foods worldwide [13].

Over the past 40 years, high-fructose corn syrup has seen substantial increases in both production and consumption [23]. The annual per capita intake of HFCS rose roughly 125% from 1970 to 1997. Arguments for such increases include HFCS’s low production cost and its ability to mix well with a variety of foods. High-fructose corn syrup is also considered part of the FODMAP family. FODMAPs are a group of short-chain carbohydrates which are often poorly absorbed in the gastrointestinal tract of susceptible individuals. These different carbohydrates were grouped together based on the length of their carbohydrate chains [24].

### 5.3. Adverse Effects of HFCS on Health

The elevated chronic consumption of HFCS has been linked to various health problems, including diabetes mellitus, non-alcoholic fatty liver disease, aging, cholesterol, and IBS.

#### 5.3.1. Hypertension

There are only a few studies in human subjects that elucidate the underlying mechanism of the adverse effects of HFCS leading to hypertension, but it has been shown that beverages containing HFCS acutely increase blood pressure. At present, the mechanisms underlying fructose-induced hypertension have not been fully characterized. Animal studies have shown that high-fructose diets upregulate sodium and chloride transporters, leading to salt overload which, in turn, increases blood pressure. Excess fructose has also been found to activate vasoconstrictors, inactivate vasodilators, and over-stimulate the sympathetic nervous system [25].

#### 5.3.2. Diabetes Mellitus

There is no causal evidence that dietary fructose causes diabetes. It appears that moderate consumption of fructose does not significantly affect fasting and non-fasting glucose levels [23]. A high intake of fructose, particularly when combined with glucose can, to a larger extent than a similar glucose intake, lead to metabolic changes in the liver; for example, increased de novo lipogenesis, and, thus, altered blood lipid profile. High fructose intake may lead to insulin resistance [26].

#### 5.3.3. Obesity

Both plasma insulin and leptin are involved in the long-term regulation of energy homeostasis. Fructose does not stimulate insulin secretion from pancreatic β cells. Thus, the production of insulin following the consumption of foods and beverages with high fructose is lower as compared to that by the consumption of glucose. This reduced level of insulin also results in low level of leptin as leptin production is regulated by insulin. The combined effect of lowered circulating leptin and insulin can, thus, increase the likelihood of weight gain. In addition, fructose, compared with glucose, is preferentially metabolized to lipids in the liver. A study showed that the consumption of fructose at 25% of energy requirements for 10 weeks, compared with isocaloric consumption of glucose, contributed to the development of metabolic syndrome by increasing circulating uric acid and liver enzymes gamma-glutamyl transferase (GGT) activity. This suggested changes in hepatic function and altered production of adipokine retinol binding protein-4 (RBP-4) [27,28].

#### 5.3.4. Non-Alcoholic Fatty Liver Disease

Non-alcoholic fatty liver disease (NAFLD) is associated with obesity and insulin resistance and is a predisposing factor for type-2 diabetes. It is characterized by increased intrahepatic fat and mitochondrial dysfunction. Excessive fructose intake may play a role in its etiology. A study showed that HFCS-55 causes downregulation of the insulin signaling pathway [29].

#### 5.3.5. High Cholesterol (Hypertriglyceridemia)

Consumption of HFCS has been shown to increase postprandial triglycerides, LDL-cholesterol, and apolipoprotein-B [30].

#### 5.3.6. Accelerated Aging

Reducing sugars, such as fructose and glucose, react with proteins and amino acids to form substituted amino sugars. This is called as glycosylation. Products of this reaction can undergo further reactions and rearrangements and accumulate on collagen and DNA. This ultimately plays a role in aging. Fructose is much more reactive than glucose with respect to participation in glycosylation. Thus, the large percentage increases in serum fructose concentrations that occur after ingestion of fructose or sucrose may have clinical consequences [23].

#### 5.3.7. IBS

IBS symptoms are triggered by the consumption of HFCS. On reaching the distal small intestine and colon, fructose increases the osmotic pressure in the large-intestine lumen and provides a substrate for bacterial fermentation, with consequent gas production, abdominal distension, and abdominal pain [31]. A study showed that almost half of the patients with IBS developed one or more symptoms upon ingestion of HFCS-55 (40 g of fructose). This level of fructose can be obtained by ingesting approximately two 12 oz cans of regular soda. The prevalence of fructose malabsorption would presumably be higher if HFCS-90 were used as the sugar source instead as fructose malabsorption is both dose- and concentration-dependent [15]. Several clinical trials were carried out to assess the effect of different forms and amounts of fructose on the development of gastrointestinal symptoms [32]. These trials emphasized the need to determine the frequency of intolerance to HFCS-55, in which the content of free fructose exceeds that of glucose, using both breath testing and evaluation of the symptoms.

## 6. Management of IBS

### 6.1. Dietary Interventions

Modification of diet is one of the most commonly used interventions for patients suffering with IBS [33]. Fiber was considered a main therapeutic approach for IBS for a long time, although the mechanism of action is unknown. Fiber’s beneficial effects may reflect colonic fermentation with production of short-chain fatty acids or its action as a prebiotic [34]. Significantly restricted diets, termed as elimination diets, are also one of the approaches used in this aspect. Improvement of symptoms was noted in a clinical trial that included 25 patients. These patients followed a strict elimination diet consisting of distilled or spring water, one meat, and one fruit for one week. Two thirds of patients who completed this diet noted symptom improvement followed by a worsening of symptoms when suspect foods were reintroduced [35]. This diet is not ideal as there is not enough evidence about its utility. There is also a risk for nutritional deficiencies. Another study addressed the potential issue of IgG related food symptoms in a randomized, blinded, controlled trial of three months’ duration. Patients with IBS who received a diet excluding foods to which they had increased IgG antibodies showed improvement in symptoms [36]. A significant improvement in stool frequency and consistency, along with relief of abdominal pain, was observed in 17 patients that adapted a very-low-carbohydrate diet (4% calorie contribution) for four weeks [37]. In another study, patients receiving low fructose/fructan diets showed improvement in all IBS related gastrointestinal symptoms such as abdominal pain, gas, bloating, nausea, diarrhea, and constipation [14]. On the other hand, diets containing low amounts of gluten, or those which were devoid of gluten, did not have marked influence in improvement of IBS symptoms in several trials. In a controlled, cross-over study of patients with IBS, a diet low in FODMAPs effectively reduced functional gastrointestinal symptoms [38]. Quite a few clinical trials have been carried out which emphasize the effectiveness of low FODMAP diets in relieving the symptoms in patients suffering from IBS [19].

Problems associated with manipulations in diet include (i) nutritional inadequacy; (ii) higher cost of restrictive diets; (iii) negative influences on socialization; (iv) development of eating disorders, particularly among patients who obsess about food and avoid certain foods; and (v) changes in gut microbiota with hitherto health implications [8].

### 6.2. Pharmacological Interventions

**Figure 1:**
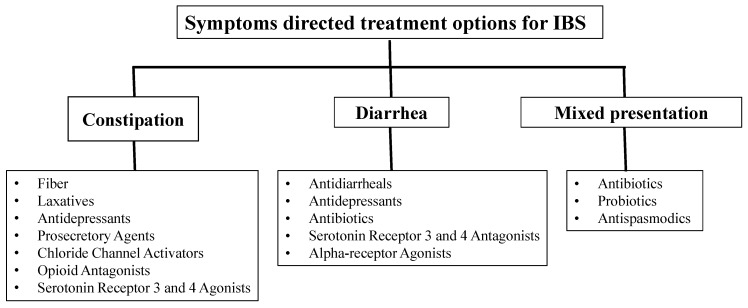
Treatment options for IBS based on symptoms. Treatment options for IBS are based on the symptoms such as constipation, diarrhea and mixed presentation of constipation and diarrhea.

IBS presents as a complex of symptoms with pathophysiological consequences ranging from intestinal inflammation to psychological dysfunction. Treating the various symptoms of IBS is a challenge and drug targets vary. Peripherally-acting treatment options for IBS are based on the predominant symptoms and IBS subtype. Existing drug therapies are administered based on a diagnosis of either constipation-predominant IBS or diarrhea-predominant IBS. Treatment options for IBS are based on the symptoms as shown in Figure 1.

Table 2 illustrates different categories of the major drugs used for the treatment of IBS, along with their mechanism of action, side effects, and benefits [5,39].

Major recent advancements in drugs to treat chronic constipation or constipation-predominant IBS fall in the general categories of 5-HT4 receptor agonists, chloride channel activators, and guanylate cyclase C receptor agonists. Recent advances in the pharmacological treatment of diarrhea-predominant IBS come from drugs that fall in the categories of opioid receptor modulators, 5-HT3 receptor antagonists, and bile acid modulators. Agonists of the 5-HT4 receptor accelerate colonic transit leading to the improvement of constipation symptoms. The successful treatment of constipation-predominant IBS with 5-HT4 agonists has been mixed. Tegaserod, a partial agonist at the 5-HT4 receptor, marketed for short term treatment of IBS, was withdrawn recently due to cardiovascular complications. Another related compound prucalopride recently demonstrated superior efficacy and generally minor side effects, headache being the most common [40]. However, in a recent clinical trial the efficacy of prucalopride was not statistically significantly different relative to a placebo [41]. The 5-HT4 receptor agonist renzapride has also been studied recently, however, in two controlled trials, a slight, or no, increase relative to a placebo combined with a possible risk of ischemic colitis has precluded further study [42,43]. In another randomized, placebo-controlled trial of patients with constipation, the 5-HT4 receptor agonist YKP10811 significantly accelerated colonic transit and led to a looser stool consistency without serious side effects [44]. Velusetrag and naronapride are both 5-HT4 receptor agonists that have been studied and have shown to increase colonic transit and the number of weekly complete spontaneous bowel movements [45].

Lubiprostone is a chloride channel type-e activator that triggers intestinal chloride secretion and sodium and fluid transit into the lumen. Lubiprostone is FDA approved for chronic constipation, based on earlier trials. In a recent placebo-controlled phase 3 trial of patients with chronic idiopathic constipation, lubiprostone was effective in improving spontaneous bowel movements, providing additional proof of efficacy [46].

Linaclotide is a peptide guanylate cyclase C receptor agonist that increases luminal chloride and fluid secretion through the generation of cyclic GMP [47]. It is currently approved for constipation-predominant IBS. Recent reports demonstrate that Linaclotide improved severe abdominal symptoms, bowel movement frequency, and was effective in improving overall quality of life [48,49]. Another guanylate cyclase C receptor agonist, Plecanatide, is under investigation for constipation-predominant symptoms, and demonstrated effectiveness in loosening stool and improving the number of spontaneous bowel movements [50].

Opioid receptor modulators are the most well-studied pharmacotherapies for diarrhea-predominant IBS, and the peripherally-acting µ-opioid receptor agonist loperimide has long been approved as an antidiarrheal agent. Recently the FDA approved the μ- and κ-opioid receptor agonist and δ-opioid receptor antagonist eluxadoline for the treatment of diarrhea-predominant IBS. It is thought that the µ-opioid receptor decreases abdominal pain and gastrointestinal propulsion, while the δ-opioid receptor prevents over-inhibition. In a double-blind, placebo-controlled trial eluxadoline treatment resulted in decreased incidence of diarrhea, as well as an analgesic effect. The most common side effects included nausea, vomiting, and abdominal pain [51]. The κ-opioid receptor agonist asimadoline was shown previously to be effective in improving pain scores, urgency, and stool frequency [52] and was recently approved by the FDA following phase 3 trials.

In a recent open label clinical trial for diarrhea-predominant IBS, the 5-HT3 receptor antagonist ramosetron demonstrated improved stool consistency and compact stools relative to a placebo, although the overall incidence of reported constipation was not significantly higher with ramosetron [46]. In this study, no serious adverse events were reported making ramosetron a promising candidate in this class for management of diarrhea-predominant symptoms

Bile acid sequestrants bind to luminal bile acids, impeding their reabsorption and reducing colonic transit. These agents may be effective in patients with diarrhea-predominant IBS, as bile acid diarrhea is due either to increased bile acid synthesis or impaired reabsorption [53,54]. Colesevelam is a bile acid sequestrant that, in an open label trial, showed evidence of intraluminal binding of bile acids, a compensatory increase in hepatic synthesis of bile acids, and improved stool consistency. The number of bowel movements per week is significantly related to the total bile acid sequestered into the stool [55].

## 7. Biopsychosocial Aspects of IBS

The physiological aspects of the IBS relate to neurologic and gastrointestinal phenomena. The impetus for the HFCS consumption and psychosocial factors compound these issues. Thus, biopsychosocial factors, particularly socioeconomic status, sex, race, and quality of life, should be considered for diagnostic evaluation of these patients.

Fruit and vegetable consumption in low-income areas is associated with low availability and access. In addition, as income decreases, consumption of added sugar increases. Although increasing income alone does not predict improved nutrition status; higher income can be a factor in increasing access to more food options. For example, the ability to drive to supermarkets and the ability to purchase healthier foods are examples of possible benefits. The combination of low income and poor diet are not novel revelations, however, for individuals susceptible to IBS, such a combination can be a cause for concern.

There is no direct link between elevated levels of HFCS consumption and a precipitated diagnosis of IBS. However, in individuals predisposed to IBS, HFCS consumption may exacerbate symptoms. There is a possible correlation among sex, added-sugar consumption, and IBS. When stratified for income, males were found to consume more added sugars than females. In 2005, the National Health Interview Survey gathered data on approximately 28,947 people regarding their added sugar intake [56]. The highlights from this observation study include: (i) in families earning less than 200% of the federal poverty level, on average, males consumed 19.2 teaspoons of sugar per day, while females consumed 13.3 teaspoons of sugar per day; (ii) in families earning between 200–399% of the federal poverty level, on average, males consumed 18.8 teaspoons of sugar per day while females consumed 12.8 teaspoons per day; (iii) in families earning greater than 400% of the federal poverty level, on average, males consumed 18.1 teaspoons of sugar per day, while females consumed 12.0 teaspoons per day. Interestingly however, females present with more clinical cases of IBS than their male counterparts.

The epidemiology of IBS among races has not been extensively studied. One study showed that, overall, non-Hispanic whites present with more cases of IBS than their non-Hispanic black counterparts. In this study, they measured a number of variables including age, sex, marital status, household income, education, and location of residence, and observed that whites presented with more cases of IBS than blacks [57].

The effects of IBS on the quality of life of the patients are negative and far-reaching. A study was carried out with a population of African-Americans residing in low-income neighborhoods using a 36-item quality of life survey. It showed that for all measures, including social, emotional, and physical functioning, African-American patients with IBS scored lower than their healthy counterparts. Patients with IBS often report increased pain sometimes severe enough to result in absence from work or school. Given the psychosocial interplay of the syndrome, treating its gastrointestinal symptoms must be matched with psychological therapy as well. Both anxiety and depression have each been acknowledged as co-morbidities of IBS [58,59]. The study also showed that those with IBS, particularly African-American females, were considerably more likely to suffer from post-traumatic stress disorder (PTSD) and suggested that evaluation of minorities presenting with functional gastrointestinal disorders should include screening for PTSD [60].

Those living in lower socioeconomic conditions have high access to processed foods and, presumably, consume higher amounts of HFCS as well. In addition, those predisposed to IBS are not only at greater risk to manifest symptoms, but may also either have a history of mental health issues or may develop mental health issues. To combat the exacerbation of symptoms, in addition to treating IBS for its psychological and gastrointestinal features, more study on the environment and diet of vulnerable populations is necessary.

## 8. Conclusions

Our medical school and hospital are located in Camden, New Jersey, which is considered a socio-economically-deprived area. The unemployment rate is double than that for the entire country, with job growth percentages being in the negative numbers. The median household income ($25,042) is less than half of the average household income ($54,462) for the entire country. Approximately 12% and 9% of the population under the age of 65 years is without insurance and is disabled, respectively. The highest-attained educational level in Camden for high school or equivalent is 67.7% and a bachelor degree of 8%. This is compared to the national average for high school of 88% and 33% for a bachelor degree. Lastly, according to US Census data, the racial breakdown in Camden is Black (48.1%), Hispanic (47%), Caucasian (17.6%), and Asian (2.1%). Although there is clearly an increased consumption of HFCS in the United States, and there is an association of HFCS with IBS, there is no published data regarding HFCS consumption in the population of Camden and its association of IBS. However as a practicing gastroenterologist in this area, the observation is that this underserved population gravitates to readily-available and inexpensive products that contain HFCS. The available treatments of dietary avoidance, as with what is used in the FODMAP diet, largely depend on patient commitment and adherence to restrictive and expensive diets. When patients are able to consume HFCS-free foods, their symptoms of IBS markedly improve. A future direction of research would be to collect data regarding HFCS consumption in our patient population and incidence of IBS, and to analyze if there is a direct correlation between the two.

## Figures and Tables

**Table 1:** Foods containing high amounts of fructose.

Food/Ingredient	Examples
Fruit	Apples, pears, clingstone peaches, mango, sugar snap peas, watermelon, cherries
Vegetables	Asparagus, artichokes, sugar snap peas
Sweeteners	Fructose, high-fructose corn syrup (sodas and processed foods), honey
Fructose	Concentrated fruit sources, dried fruit, fruit juice

**Table 2:** General pharmaceutical treatment options for IBS.

Drug Class	Mechanism	Side Effects	Benefit
**Antidiarrheals**	Increase GI transit time and decrease secretion via peripheral u-opioid receptors; reduce visceral afferent pathway inhibition	May exacerbate constipation	Loperamide reduces abdominal pain and fecal urgency
Loperamide		Blurred vision	
		Vomiting	
Diphenoxylate/Atropine		Diarrhea	
		Nausea	
**Antidepressants: TCA**	NE and 5HT reuptake inhibitor at neuronal membrane; may down-regulate b-adrenergic and serotonin receptors	May exacerbate GI symptoms	Reduce visceral nocioception via afferent pathway inhibition
Imipramine			Prolong GI transit; decrease secretion
Amitryptyline			
**Antibiotics**	Change bacterial content in GI tract and reduces gas	Headache	Prevents bacterial overgrowth
Rifaximin	Binds to b-subunit of DDRP to inhibit transcription	Rectal tenesmus	
		Abdominal pain	
**Serotonin Receptor 3 Antagonists**	Inhibit receptors on myenteric, splanchnic, and vagal nerves	Ischemic colitis with alosetron	Reduces colonic hypersensitivity and gut motility
Alosetron		Constipation	
Cilansetron			
**Antispasmodics/**	Peppermint oil possesses calcium channel blocking properties	Reflux	Inhibits parasympathetic activity in smooth muscle, secretory glands, and CNS
**Anticholinergics**	Inhibition of ACh at receptor		
Peppermint Oil			
Dicyclomine Hydrochloride			
Hyoscyamine Sulfate			
